# Complete Genome Sequence of Enterotoxigenic *Escherichia coli* Myophage Murica

**DOI:** 10.1128/genomeA.01135-15

**Published:** 2015-10-01

**Authors:** Joseph N. Wilder, Jacob C. Lancaster, Jesse L. Cahill, Eric S. Rasche, Gabriel F. Kuty Everett

**Affiliations:** Center for Phage Technology, Texas A&M University, College Station, Texas, USA

## Abstract

Murica is an rv5-like myophage that infects enterotoxigenic *Escherichia coli*. Pathogenic *E. coli* strains are responsible for many intestinal diseases, and phages that infect these bacteria may prove useful in preventing severe health issues. The following is a report of the complete genome sequence of Murica and its important features.

## GENOME ANNOUNCEMENT

Enterotoxigenic *Escherichia coli* strains serve as one of the leading causes of moderate to severe diarrhea in developing countries, affecting more people than *Shigella* or *Salmonella* species in many areas (1–3). The U.S. Food and Drug Administration has reported that bacteriophages are safe for use as a food additive in some meat and poultry products, paving the way for the production and use of phage-based products against enterotoxigenic *E. coli* (4). Here, we describe the complete genome sequence of a novel rv5-like myophage, Murica, which is active against enterotoxigenic *E. coli*.

Bacteriophage Murica was isolated from a pig fecal sample collected at College Station, TX. Phage DNA was sequenced in an Illumina MiSeq 250-bp paired-end run with a 550-bp insert library at the Genomic Sequencing and Analysis Facility at the University of Texas (Austin, TX). Quality controlled trimmed reads were assembled to a single contig of circular assembly at 86.6-fold coverage using SPAdes version 3.5.0 (5). The contig was confirmed to be complete by PCR using primers that face the upstream and downstream ends of the contig. Products from the PCR amplification of the junctions of concatemeric molecules were sequenced by Sanger sequencing (Eton Bioscience, San Diego, CA). Genes were predicted using GeneMarkS (6) and corrected using software tools available on the Center for Phage Technology (CPT) Galaxy instance (https://cpt.tamu.edu/galaxy-public/). Morphology was determined using transmission electron microscopy performed at the Texas A&M University Microscopy and Imaging Center.

Murica contains a 135,391-bp genome that has a coding density of 90.7%. It has a G+C content of 43.6%, which is lower than the average G+C content for enterotoxigenic *E. coli* strains (50%) (7). The genome contains 212 putative coding sequences, 49 of which code for predicted functions based on InterProScan and BLASTp analysis (8, 9). Murica shares 91.5% nucleotide sequence identity across the genome with *E. coli* myophage rv5, as determined by Emboss Stretcher (10). It is a member of the Lytic22 cluster recently described by Grose and Casjens (11). Murica was opened to the *rIIa* gene in accordance with the precedent set by rv5. Seven tRNA genes were identified compared to the five found in rv5 (12).

Murica has a 2,554-bp region that is comparable to the 2,604-bp noncoding region in rv5; however, the gap in Murica appears to contain a novel gene of unknown function. Rv5 has an HNH homing endonuclease interrupting the large terminase, while the large terminase remains intact in Murica. The tail fiber protein (Murica36) shows 99% amino acid identity with the tail fiber protein of *E. coli* O157:H7 phage vB_EcoM_FFH2 (accession no. NC_024134), a phage that has been described as having broad host range and was recently shown to be effective in reducing *E. coli* O157:H7 contamination in spinach and beef (13, 14). This suggests that Murica may also have a broad host range and may be useful in the treatment of contaminated food products.

### Nucleotide sequence accession number.

The genome sequence of phage Murica was contributed as accession no. KT001917 to GenBank.
